# Hypoxia Performance Curve: Assess a Whole-Organism Metabolic Shift from a Maximum Aerobic Capacity towards a Glycolytic Capacity in Fish

**DOI:** 10.3390/metabo11070447

**Published:** 2021-07-08

**Authors:** Yangfan Zhang, Bog E. So, Anthony P. Farrell

**Affiliations:** Department of Zoology, Faculty of Land and Food System, University of British Columbia, Vancouver, BC V6T 1Z4, Canada; so.yuliya@gmail.com (B.E.S.); tony.farrell@ubc.ca (A.P.F.)

**Keywords:** hypoxia, maximum oxygen uptake, standard metabolic rate, P_crit_, P_50_, metabolism, limiting-O_2_ concentration performance curve, respiratory physiology, ecological physiology, comparative physiology

## Abstract

The utility of measuring whole-animal performance to frame the metabolic response to environmental hypoxia is well established. Progressively reducing ambient oxygen (O_2_) will initially limit maximum metabolic rate as a result of a hypoxemic state and ultimately lead to a time-limited, tolerance state supported by substrate-level phosphorylation when the O_2_ supply can no longer meet basic needs (standard metabolic rate, SMR). The metabolic consequences of declining ambient O_2_ were conceptually framed for fishes initially by Fry’s hypoxic performance curve, which characterizes the hypoxemic state and its consequences to absolute aerobic scope (AAS), and Hochachka’s concept of scope for hypoxic survival, which characterizes time-limited life when SMR cannot be supported by O_2_ supply. Yet, despite these two conceptual frameworks, the toolbox to assess whole-animal metabolic performance remains rather limited. Here, we briefly review the ongoing debate concerning the need to standardize the most commonly used assessments of respiratory performance in hypoxic fishes, namely critical O_2_ (the ambient O_2_ level below which maintenance metabolism cannot be sustained) and the incipient lethal O_2_ (the ambient O_2_ level at which a fish loses the ability to maintain upright equilibrium), and then we advance the idea that the most useful addition to the toolbox will be the limiting-O_2_ concentration (LOC) performance curve. Using Fry & Hart’s (1948) hypoxia performance curve concept, an LOC curve was subsequently developed as an eco-physiological framework by Neil et al. and derived for a group of fish during a progressive hypoxia trial by Claireaux and Lagardère (1999). In the present review, we show how only minor modifications to available respirometry tools and techniques are needed to generate an LOC curve for individual fish. This individual approach to the LOC curve determination then increases its statistical robustness and importantly opens up the possibility of examining individual variability. Moreover, if peak aerobic performance at a given ambient O_2_ level of each individual is expressed as a percentage of its AAS, the water dissolved O_2_ that supports 50% of the individual’s AAS (DO_AAS-50_) can be interpolated much like the P_50_ for an O_2_ hemoglobin dissociation curve (when hemoglobin is 50% saturated with O_2_). Thus, critical O_2_, incipient lethal O_2_, DO_AAS-50_ and P_50_ and can be directly compared within and across species. While an LOC curve for individual fish represents a start to an ongoing need to seamlessly integrate aerobic to anaerobic capacity assessments in a single, multiplexed respirometry trial, we close with a comparative exploration of some of the known whole-organism anaerobic and aerobic capacity traits to examine for correlations among them and guide the next steps.

## 1. Introduction

Animal life is sustained by a tightly regulated oxidization of metabolic substrates to support ATP production. An animal’s oxygen (O_2_) transport cascade system conceptualizes the movement of O_2_ down its tension gradient from the ambient environment (160 mm Hg at sea level) to the mitochondria (<1 mm Hg), where O_2_ is consumed to generate ATP. Hence, if the ambient environment becomes hypoxic, the gradient of O_2_ tension (PO_2_) decreases which, at some point, limits the maximum O_2_ flux through the O_2_ transport cascade. Thus, by constraining maximum aerobic capacity, hypoxia can potentially impact an animal’s lifetime fitness, as has been seen when supra-optimal temperature constrains the maximum aerobic capacity of adult migrating sockeye salmon [1] and limits their ability to reach their natal spawning areas [2,3].

Environmental hypoxia is a pressing issue. For example, our oceans have lost 150 trillion kilograms of O_2_ in the last 50 years [4], with the largest loss (40%) being manifest as large O_2_-minimum zones (<80 μmol kg^−1^ O_2_ within the water column) in the North and Equatorial Pacific Oceans [5]. To answer whether or not our current hypoxic trajectory will mean a pessimistic outlook for fishes, we will need a much better understanding of the standing individual variation of hypoxic performance across fish species. Furthermore, this level of understanding will require tools that can assess the respiratory phenotype of fishes well beyond those we routinely use. For instance, while the Permian era, oceanic deoxygenation wiped out an estimated 96% of marine fishes [6], we now know that after the subsequent reoxygenation of the oceans teleost fishes successfully radiated into almost every aquatic habitat on the planet aided by the evolution of new and very proficiency modes of tissue oxygenation [7].

Fish employ a repertoire of compensatory responses during hypoxia to defend their O_2_ supply to essential tissues [8,9,10,11,12]. For example, moderate hypoxia in the short term can trigger hyperventilation [13,14], increase blood perfusion of gill lamellae [15], induce bradycardia which increases the time for O_2_ diffusion at gill lamellae [16,17,18,19], increase venous tone which improves the return of blood to the heart [20,21], induce the splenic release of red blood cell stores which increases the blood O_2_ carrying capacity [22,23] and prioritize blood flow to essential tissues [24,25]. Many of these responses are aimed at preventing a hypoxemic state, a reduced arterial blood O_2_ content (C_a_O_2_). If this is not possible and C_a_O_2_ falls, both venous O_2_ content (C_v_O_2_) and maximum O_2_ uptake (*Ṁ*O_2max_) will eventually also decrease because fish rarely tap their scope to increase cardiac output in hypoxia [12].

Chronic exposure to hypoxia can establish new respiratory phenotypes in some fish species, ones that improve tolerance of hypoxia. For example, they can resort to a hypometabolic state by temporarily turning off some basic activities and lowering basal tissue O_2_ demand, a response manifested by some species that can tolerate the most severe form of hypoxia—anoxia [26]. Also, they can remodel certain tissues, including the gills [27,28,29], the heart [30,31] and the swimming muscles [32] to better tune the O_2_ cascade to a reduced O_2_ gradient. Obviously, knowledge of these adaptive strategies and mechanisms will be crucial to know which fish species might have an evolutionary edge in a more hypoxic future, but how best to holistically measure hypoxic performance traits in fishes is less clear. We believe that the situation can be improved by adopting concepts, techniques and analytical tools that have improved measurements of thermal performance curves in fish, an area that has received considerable attention recently (see reviews: [33,34]).

Currently, the two most widely used whole-organism hypoxic performance traits for eco-physiological studies are the critical dissolved O_2_ (O_2crit_) and the incipient lethal O_2_ saturation (ILOS). Both measurements employ well-established techniques and can be performed under field conditions. O_2crit_ is the ambient dissolved O_2_ (DO) level below which maintenance metabolism (i.e., standard metabolic rate, SMR) cannot be sustained by oxidative phosphorylation and is supplemented by substrate-level phosphorylation. ILOS is the DO level at which a fish loses the ability to maintain upright equilibrium and represents an ambient DO that can be tolerated only for a limited amount of time, an ILOS that is probably close to a point of no return. O_2crit_ and ILOS mark the DO range for Hochachka’s concept of scope for survival, a term coined to characterize time-limited life when SMR cannot be supported by O_2_ supply. 

While the comparative values of both O_2crit_ and ILOS are well established because they clearly vary across species, among strains and populations within a species and even among individuals, they can also show compensatory changes after hypoxic acclimation, but their ecological relevance has been questioned. Therefore, we need tools that assess hypoxic performance beyond just O_2crit_ and ILOS. Sub-lethal performance curves, ones that can identify biological safety margins for an animal’s activities, maybe the next step forward. For example, sub-lethal toxicity tests have replaced lethality tests and sub-lethal performance measures are similarly being routinely used to assess the thermal performance of fishes. The key question we address in this review is which whole-organism performance traits might better quantify the sub-lethal effects of hypoxia? We believe an individualized hypoxia performance curve in the form of a limiting-O_2_ concentration (LOC) performance curve for maximum metabolic rate (MMR) is a crucial first step. An individual-based and standardized experimental approach would be fundamentally different from a break-point analysis of the maximum capability to uptake O_2_ under normoxic and hypoxic conditions using studies that might have employed different measurement protocols [35]. But before we introduce the individual LOC curve, we briefly review the ongoing debate concerning the importance of standardizing methodologies when assessing a fish’s hypoxic performance.

## 2. Current Hypoxia Tolerance Assessments Explore Only a Small Portion of Hypoxic Performance Curve

A widely-used landmark of hypoxic performance is the minimum amount of ambient O_2_ needed by a fish to aerobically sustain its whole-organism maintenance metabolic demands, i.e., O_2crit_ (% sat.), the term we use generically for this article. This ambient O_2_ threshold can also be measured as the critical O_2_ tension (P_crit_; mm Hg) and critical O_2_ concentration (C_crit_; mg L^−1^), each of which has certain benefits (for example, water O_2_ saturation is dependent on ambient atmospheric pressure and temperature; P_crit_ is dependent on ambient atmospheric pressure but independent of temperature except for the effect on water vapour pressure; C_crit_ is dependent on ambient atmospheric pressure and temperature).

Original studies used SMR to define O_2crit_, in part because, by definition, a fish has no aerobic scope at O_2crit_ [10,11,36]. However, debate continues concerning the use of routine metabolic rate (RMR) as the baseline for determining O_2crit_ (e.g., [37,38]). Some have argued that an RMR-derived O_2crit_ has a greater ecological relevance because it incorporates the metabolic cost of ‘idling’ in a wild fish that would spend little, if any, time at SMR in nature [39]. Mueller and Seymour (2011) also advanced the Regulatory Index, an area under routine or active O_2_ uptake (*Ṁ*O_2_) versus DO curve above a linear trend of perfect conformity, with a larger area indicating a greater degree of regulation [40]. RMR, however, is a highly variable baseline, one that would be difficult to replicate from lab-to-lab and fish-to-fish. Levels of spontaneous activity are difficult to quantify without directly monitoring or controlling activity, although some earlier studies used drugs to immobilize fish (*see* [41]). In contrast, SMR is a less ambiguous baseline that can be reliably estimated using established testing apparatus, protocols and analytical tools provided *Ṁ*O_2_ in undisturbed fish is continuously measured over at least 48 h (e.g., [41,42,43]). Thus, a recent consensus among fish biologists is that a SMR-derived O_2crit_ represents a robust analytical approach with a stable baseline that increases the repeatability of O_2crit_ measurements and the utility of O_2crit_ for comparisons [44,45,46,47]. Whether or not SMR has ecological relevance will be an ongoing debate. 

**Figure 1:**
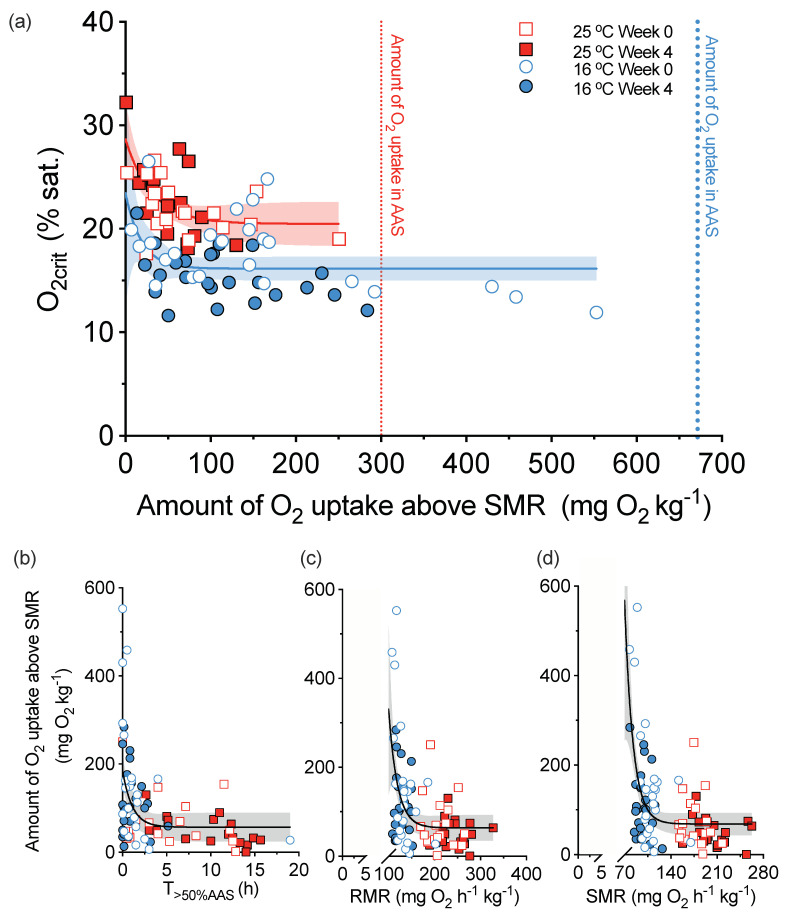
The interdependence of critical dissolved oxygen (O_2crit_), behavioural activity, and standard metabolic rate (SMR) in European sea bass (*Dicentrarchus labrax*). Fish were acclimated to either 16 or 25 °C, and the same fish from each acclimation temperature were repeatedly measured after 4 weeks to assure the reproducibility of the first measurement. The activity level in hypoxia was measured by the amount of oxygen (O_2_) uptake above SMR during a hypoxia challenge test. Behavioural activity in normoxia was measured by the routine metabolic rate (RMR) and time spent above 50% absolute aerobic scope (T > 0.5%AAS). The amount of O_2_ uptake in absolute aerobic scope (AAS) is what fish would consume if using a full AAS in the hypoxia challenge test. The non-linear regression analyses used a one-phase association model. Panel (**a**) explores how activity in hypoxia can affect O_2crit_. Panels (**b**–**d**) explore if the sea bass showed a consistent behavioural activity pattern in normoxia and hypoxia. The figure and data are adapted from [51].

All the same, activity can influence O_2crit_ even when SMR is used as a baseline. For example, a higher activity during hypoxia correlated with a lower O_2crit_ in European sea bass (*Dicentrarchus labrax*) until the activity measured by the amount of O_2_ consumption above SMR was higher than 100 mg O_2_ kg^−1^ during hypoxia exposure (Figure 1a). The more active fish in hypoxia are, however, the calmer fish in normoxia and have a lower SMR (Figure 1b–d). Thus, the debate will be ongoing as to whether or not such relationships relate to a pace-of-life syndrome, wherein individuals of higher metabolism generally exhibit more active behaviours and physiological traits than their conspecifics with a lower metabolism [48,49].

**Figure 2:**
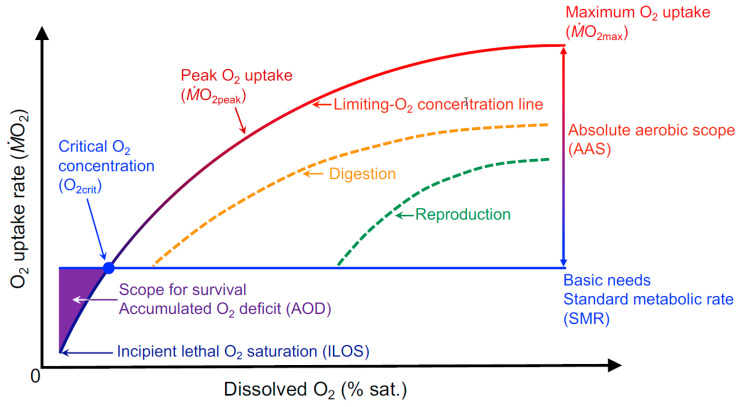
A conceptual representation of the oxygen (O_2_) needs and capacities for supply in a fish as a function of the dissolved O_2_ in the water (% sat.). The various curves and their placement acknowledge and unify several concepts already advanced in the literature. The upper bounding limiting-O_2_ concentration (LOC) line for peak O_2_ uptake (*Ṁ*O_2_) (which is maximum O_2_ uptake, *Ṁ*O_2max_, under normoxic conditions) and those for digestion and reproduction are based on the conceptual framework for hypoxic performance curves [11,25,36], and the experimental LOC lines generated for groups of fish [52,53]. An LOC performance curve, therefore, bounds and sets the capacity for the potential use of O_2_ for all other activities, as similarly suggested beyond the pejus temperature for warming [54], potentially including a buffer in O_2_ availability before an activity is constrained [1]. Exactly how O_2_ is apportioned amongst the various activities and what governs the apportioning are unknown, and so the shape and positioning of the broken lines are unknown. We define O_2crit_ as a minimum amount of ambient O_2_ concentration at which a fish can aerobically sustain its whole-organism minimum maintenance metabolic demands, i.e., its standard metabolic rate (SMR) [10,46]. Thus, below O_2crit_, *Ṁ*O_2_ conforms in some manner with O_2_ availability and ATP needs for survival until incipient lethal O_2_ saturation (ILOS; where death is imminent) must be met through a combination of oxidative phosphorylation, substrate-level phosphorylation, and possible suppression of some aspects of maintenance metabolism. Hochachka [55] coined the term scope for survival as the difference between SMR and the lowest sustainable rate to which metabolism may be suppressed before reaching ILOS. As an index of the scope for survival, we calculate accumulated O_2_ deficit (AOD) as the integral of the area bounded by SMR and the *Ṁ*O_2_ measured between O_2crit_ and ILOS.

Beyond a reliable measurement of SMR in normoxia to set the baseline for determining O_2crit_, a fish’s *Ṁ*O_2_ must be measured during a progressive exposure to hypoxia [10,46]. Routine *Ṁ*O_2_ may remain unchanged over much of the hypoxic range or increase slightly (either because the fish becomes agitated or due to an added O_2_ cost of increasing external and internal O_2_ convection), but *Ṁ*O_2_ will eventually decrease and conform to an ever-decreasing amount of available O_2_ (Figure 2). The O_2crit_ is interpolated from a slope determined by a slope of *Ṁ*O_2_ points near to when the threshold SMR (or RMR) can no longer be maintained [46]. The O_2crit_ slope was reiterated as an alpha slope, average of maximum slope from three *Ṁ*O_2_ points and is forced through the origin of the axis, to quantify O_2_ supply capacity [50]. Whether or not the alpha slope is constant across a whole range of DO remains to be tested by some rigorous studies.

The O_2crit_ protocol (or hypoxic challenge test) can be performed using either closed-flow or intermittent-flow respirometry. With closed-flow respirometry, the fish regulates the rate of induction of hypoxia and, although metabolic wastes progressively accumulate inside the respirometry chamber [56,57], this accumulation appears to have a negligible effect on the O_2crit_ estimate [58]. An intermittent-flow respirometry system offers a tighter regulation of the rate of induction of hypoxia and wastes are intermittently removed, a beneficial feature for determining an LOC performance curve.

Scope for survival [55] applies to life when ambient water O_2_ is below O_2crit_ and a fish supplements maintenance metabolism with substrate-level phosphorylation [59] and/or enters a hypometabolic state (some basic metabolic needs are temporarily switched off or turned down; *see* review by [8]. Metabolic suppression (often coupled with cold exposure) can involve arresting ion channels, spike potentials and protein translation, which are associated with inactivity, reduced cardiac activity and suspended growth [60,61,62,63]. A lethargic state is a constraint on RMR and AMR, something can be induced by cooling and darkness [64] and is fundamentally different from a metabolic suppression.

ILOS is a widely-used landmark on the hypoxic performance curve of fishes [10,65] and is the point where ambient DO is so low that a fish loses neurological control and can no longer maintain an upright equilibrium (except perhaps for fishes that have morphological structure to hold themselves upright even when they lose neurological control). ILOS, however, is quite sensitive to the rate at which ambient DO is reduced, in part, because glycogen stores are finite [66,67,68] and, in part, because the accumulation of metabolic wastes from glycolysis may eventually reach toxic thresholds. For example, exceeding the plasma lactate threshold suggested for fish (~20 mmol L^−1^; [69,70]) seems to signal delayed mortality [71,72]. Indeed, some anoxia-tolerant ectotherms have evolved extraordinary strategies to mitigate the accumulation of these metabolic wastes. For example, goldfish (*Carassius auratus*) convert lactate to ethanol, which is a H^+^-consuming reaction [73,74] and the ethanol is produced easily and diffuses out of the fish across the gills provided the circulation is maintained. Thus, despite the drawback of losing carbon energy, ethanol-producing anoxic crucian carp (*Carassius carassius*) still maintain their routine cardiac activity [74] and do not become comatose [75]. In contrast, the anoxic freshwater turtle (*Chrysemys picta*) becomes comatose for months and avoids a fatal acidosis by demineralizing carbonates from its shell while at the same time complexing the lactate ions with calcium and magnesium [76].

Scope for hypoxic survival is necessarily time-limited and typically in the range of minutes for most fishes that do not tolerate severe hypoxia. Life below O_2crit_ and above ILOS also incurs an O_2_ deficit, which can be measured with post-hypoxia O_2_ uptake measurements [77]. Alternatively, the accumulated O_2_ deficit (AOD) can be indirectly estimated from continuous *Ṁ*O_2_ measurements between O_2crit_ and ILOS if DO decreases at a steady rate. AOD is obtained by integrating the area bounded by the SMR threshold level and measured *Ṁ*O_2_ values between O_2crit_ and ILOS [78]. This AOD calculation has many assumptions, all of which need to be experimentally checked. For example, an acceptable, standardized rate of hypoxia induction is needed. Also, the possibility of a metabolic suppression leading to AOD being an overestimate of the true O_2_ deficit needs to be explored. 

Metabolic suppression is certainly possible in hypoxia-acclimated fish. For example, European sea bass (*Dicentrarchus labrax*), an athletic teleost, suppressed SMR by 20% after hypoxic acclimation [51]. A more extreme metabolic suppression has evolved in anoxia-tolerant aquatic species (*see* review by [8]) like crucian carp (*Carassius carassius*) and freshwater turtles (*Chrysemys picta bellii*) which was estimated to reduce SMR up to 90% for up to 5 months [75,79]. Bushnell et al. (1984) speculated that hypoxia-acclimated rainbow trout (*Oncorhynchus mykiss*) suppressed SMR, but to reach this conclusion they extrapolated *Ṁ*O_2_ measurements during swimming to a zero water velocity, a methodology that does not accurately measure SMR [80]. Hypoxia-acclimated steelhead trout (*Oncorhynchus mykiss*) maintained the same routine *Ṁ*O_2_ when measured in a normoxic thermal challenge [81]. Even so, a danger of using an RMR-derived O_2crit_ is that hypoxic suppression of behavioural activity could be mistaken as metabolic suppression. Likewise, using RMR rather than SMR to derive O_2crit_ could confound assessments of the time course of the onset of metabolic suppression because behavioural activity was initially suppressed rather than maintenance metabolism. Nonetheless, severe hypoxia or anoxia decreased RMR within ~15 mins in tilapia (*Oreochromis* spp.) [62], <1 h in Pacific hagfish (*Eptatretus stoutii*) [77] and goldfish (*Carassius auratus*) [82] and <1.5 h in killifish (*Fundulus heteroclitus*) [83]. While the suppression of locomotor activities cannot be ruled out as a contributing factor, the large reductions in cardiac output seen during severe acute hypoxia in common carp (*Cyprinus carpio*), tilapia and freshwater turtles [62,84,85] suggest a rapid onset of metabolic suppression in some ectotherms, especially fishes. Therefore, the time course of SMR suppression will need further investigation as to its potential effect on AOD and even O_2crit_ determinations. 

Clearly, O_2crit_ and ILOS determinations have unquestionably served fish biologists well for many decades. Yet, we predict measurement tools that identify sublethal effects of hypoxia will be more useful for conservationists and modellers of future hypoxic scenarios. To this end, Fry & Hart [10] provided a conceptual framework to holistically characterize the whole-organism function in a hypoxic environment using a hypoxia performance curve. Today, the hypoxia performance curve has taken the form of a limiting-O_2_ concentration (LOC) performance curve for MMR, something we feel should be the most useful framework and assessment tool for an immediate addition to the toolbox for assessing hypoxic performance.

## 3. Individualizing the Limiting-Oxygen Concentration (LOC) Performance Curve

The underlying concept of a LOC curve for MMR is that maximum aerobic capacity (termed peak O_2_ uptake, *Ṁ*O_2peak_, for clarity if it is not measured in normoxia) must decrease once ambient DO produces a hypoxemic state (Figure 2) and when an O_2_ diffusion limitation exists at the gills. Consequently, as *Ṁ*O_2peak_ decreases as a function of declining DO, AAS will decrease until O_2crit_ is reached and AAS is zero, as defined above. Making the required *Ṁ*O_2_ measurements to generate the LOC performance curve, however, is not a trivial task. First, there is the methodological challenge at a given DO is to assure a fish reaches a peak active state to obtain *Ṁ*O_2peak_. Then, as DO is reduced in a stepwise fashion, the peak active states need to be repeated sufficient times to accurately model the resulting curve that describes the decline in *Ṁ*O_2peak_ with DO.

The first attempts to generate a LOC performance curve involved exercising a group of fish in a respirometer at one DO and then another group at a different DO [86,87]. This approach precluded the concern of a progressive depletion or build-up of metabolites if a fish or group of fish was repeatedly chased during progressive hypoxia. LOC curves derived in this fashion have been used in fishery models [52] and eco-physiological frameworks [53]. The disadvantage of this group approach is that the pooled data for different individuals, which generate the single curve, will smooth out the LOC performance curve and it cannot characterize individual variation, the very thing that natural selection acts on. 

Rather than chasing groups of fish to generate *Ṁ*O_2peak_, fish can be swum individually in a swimming respirometer with a critical swimming speed (*U*_crit_) protocol or a shorter ramped-*U*_crit_ protocol. However, this methodological approach would be too time-consuming to repeatedly measure *Ṁ*O_2peak_ in this manner as a function of DO. First, the large water volume of the swimming respirometer takes a considerable time to equilibrate at each new DO level. Second, even a ramped-*U*_crit_ protocol takes ~1.5 h to complete. Thus, if say five DO levels were used to generate the LOC performance curve (likely the minimum number), it would take 7.5 h for completion without including the DO equilibration times. Consequently, swimming tests do not lend themselves well to an LOC curve determination.

**Figure 3:**
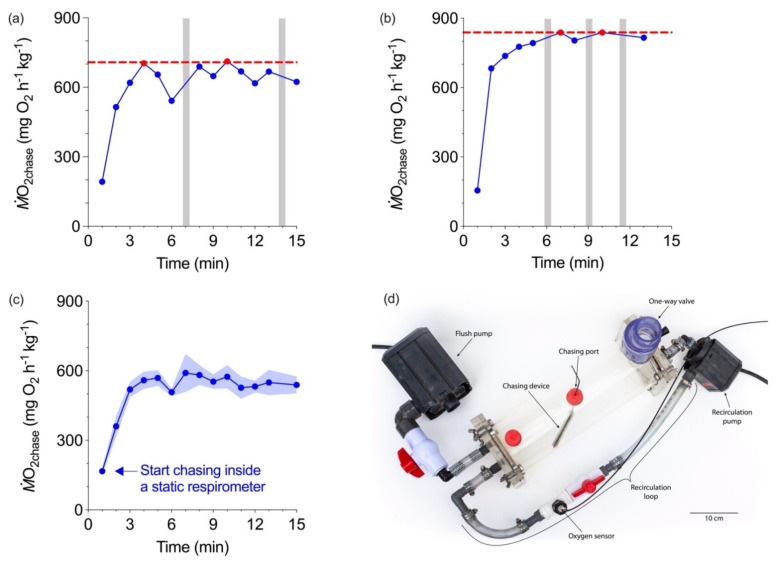
Individual traces (**a**,**b**) of continuous recordings of oxygen uptake (*Ṁ*O_2_) of a rainbow trout (*Oncorhynchus mykiss*) while it is continuously agitated for 15 min inside a respirometer (*Ṁ*O_2chase_) at normoxia (>80% air sat.). Grey vertical bars represent the flush period. Peak *Ṁ*O_2_ (*Ṁ*O_2peak_, the horizontal broken line) is based on the average of the two highest values (two red dots as duplicates), which typically occur within the first 10 min, although a fish can reach or approach this peak several times. Group average of *Ṁ*O_2peak_ for 10 fish (**c**) shows that a commonality that *Ṁ*O_2peak_ is consistently elevated. *Ṁ*O_2peak_ values were estimated in a 1 min sequential interval regression algorithm. (**d**) A modified static respirometer to monitor *Ṁ*O_2peak_ while fish is agitated to fatigue in the sealed respirometry chamber. The figure and data were adapted from [88].

*Ṁ*O_2peak_ is also measured by chasing individual fish to exhaustion outside a respirometer and then following post-exhaustion recovery of routine *Ṁ*O_2_ to measure *Ṁ*O_2peak_. An individual fish then could be exhausted at different DO levels before being placed into a respirometer at the same DO levels. This protocol could be used on different individuals at each DO level or on the same individual by repeatedly placing a fish in and out of the respirometer. The latter approach could be technically very challenging as well as unnecessarily stressful to the fish. Maintaining the chasing apparatus at the same DO as the respirometer would be another challenge. Of greater concern is that chasing a fish outside of a respirometer potentially underestimates *Ṁ*O_2max_ [88] and so this problem has been avoided by adding a simple chasing device to a standard respirometry chamber (Figure 3d). This way a fish can be agitated to generate a reliable *Ṁ*O_2peak_ [88] and even repeatedly agitated without having to remove the fish from the respirometer.

This technical modification to a respirometer now means that DO inside the respirometer can be progressively reduced between repeated exercise bouts of an individual fish inside a respirometer [65,89]. Thus, it is possible to measure *Ṁ*O_2peak_ as a function of declining ambient O_2_ and generate an LOC performance curve for individual fish [51] and using far fewer fish than in a group-LOC protocol.

This individual LOC curve protocol will need further refinements to better conform with the 3-R principles (replacement, reduction & refinement) for animal care and for quality control purposes. For example, fish was exercised for 10 min first at a DO of 95% sat. DO was decreased progressively over 125 min, *Ṁ*O_2peak_ was repeatedly determined with a 5-min agitation period every 10 min at 10 additional DO levels [51]. Protocol improvement should consider the following.

First, the duration of the chase needs to be standardized. A 10-min chase may be unnecessarily long to generate a *Ṁ*O_2peak_, which is a key requirement. Crucial to refining this will be replacing the conventional, on-line sequential interval regression analysis of *Ṁ*O_2_ with an off-line analysis of the rolling regression algorithm coupled with a minimum sampling window (typically 1–2 min) [90]. This analytical protocol can better resolve the transient nature of *Ṁ*O_2peak_ by selecting the maximum and reliable (i.e., *R^2^* > 0.98) value from all the measurements recorded during and immediately (i.e., 5 mins) after the chase. Perhaps agitation could be stopped once two or three similar peaks were seen or pilot experiments could set a reliable period of chasing to ensure fatigue set in and 2–3 *Ṁ*O_2peak_ values are recorded. Also, rather than mechanically agitating fish, it may be possible to use other stimuli to reliably generate *Ṁ*O_2peak_ such as switch off of lights [91].

Second, the number of *Ṁ*O_2peak_ measurements per fish needs to be minimized to further minimize cumulative stress. The concern here is that the build-up of metabolites such as lactate and H^+^ could limit *Ṁ*O_2peak_ beyond that of DO alone. Likewise, if muscle glycogen stores become depleted, the fish will not be able to reach *Ṁ*O_2peak_. Both of these biological issues would manifest as a right- or down-shift of the LOC performance curve. Comparing fish tested at a single DO level with those tested individually at multiple DO levels could test for this possibility. Minimizing the DO levels tested on an individual, however, runs the risk of reducing the statistical power of accurately modelling the LOC performance curve. Obviously, the LOC curve is anchored by *Ṁ*O_2max_ in normoxia (say > 90% sat.), but it does not have to be anchored by O_2crit_. In fact, O_2crit_ might be better determined in a separate protocol for that fish and even before the LOC curve protocol starts to ensure that chasing a fish beforehand does not result in an overestimated SMR. A curve or breakpoint will likely require a minimum of five DO measurements for an accurate description. Preliminary experiments could be used to strategically place these DO levels on crucial parts of the curve. Ultimately the data will determine which is the best statistical approach to model these relationships, either a curvilinear fitting (e.g., one-phase association regression model; [40]) or a rectilinear fitting (e.g., segmented linear regression model; [35]), based on the inspection of the model quality (e.g., AIC values and *R*^2^). 

**Figure 4:**
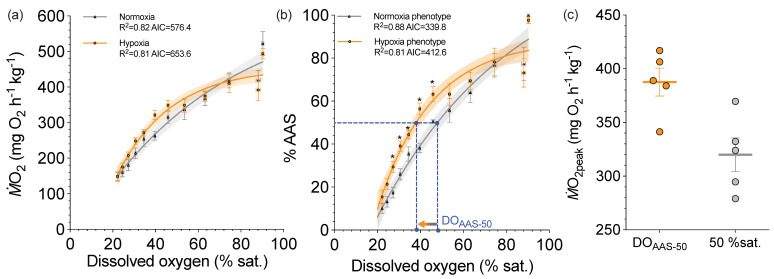
The limiting-oxygen (O_2_) concentration performance curve of peak O_2_ uptake (*Ṁ*O_2peak_) in normoxia- and hypoxia-acclimated juvenile European sea bass (*Dicentrarchus labrax*) at 16 °C. *Ṁ*O_2_ values are measured (**a**) then normalized (**b**) as a percentage of absolute aerobic scope (% AAS) in each individual. Mean ± SEM for *Ṁ*O_2_ at a given dissolved O_2_ in water in percentage air saturation (% sat.) was used to characterize a group HPC performance curve using one-phase association equations [normoxia phenotype of absolute *Ṁ*O_2_: (**a**) normoxic phenotype: y = −52.8 + (642.4 + 52.8) × [1 − e ^(−0.016 × X)^]; R^2^ = 0.82, AIC = 576.4; hypoxic phenotype of absolute *Ṁ*O_2_: y = −268.3 + (454.9 + 268.3) × [1 − e ^(−0.039 × X)^]; R^2^ = 0.81, AIC = 653.6 (**b**) normoxic phenotype of normalized *Ṁ*O_2_: y = −39.5 + (130.0 + 39.5) × [1 − e ^(−0.005 × X)^]; R^2^ = 0.88, AIC = 339.8; hypoxia phenotype of normalized *Ṁ*O_2_: y = −85.8 + (88.3 + 85.8) × [1 − e ^(−0.057 × X)^]; R^2^ = 0.81, AIC = 412.6]. The solid curves are one-phase association regression models, and the shaded areas are the 95% confidence intervals of these curves. Asterisks denote the statistical significances between arcsine transformed % AAS between normoxia- and hypoxia-acclimated fish at each dissolved O_2_ saturation (independent sample *t*-tests, *α* < 0.05). Blue dash lines illustrate the comparison of dissolved O_2_ (DO) for 50% of AAS (DO_AAS-50_). The values of DO_AAS-50_ are labelled by the blue dots on the x-axis. (**c**) Scatterplots (mean ± SEM) for the comparisons of *Ṁ*O_2peak_ measured at DO_AAS-50_ and *Ṁ*O_2peak_ measured by one bout of 10 min chase inside the respirometer at 50% air saturation (% sat.). The figure and data are adapted from [51].

Third, a metric is needed to compare the LOC performance curves. In this regard, we feel the best comparative metric may be the DO that maintains 50% AAS (DO_AAS-50_), which borrows from the idea of P_50_ of a blood O_2_ equilibrium curve. If SMR and *Ṁ*O_2max_ are known for each individual, each *Ṁ*O_2peak_ value can be expressed as a percentage of its absolute aerobic scope (AAS). Thus, an individual LOC performance curve can be fitted to 100% AAS [51], which adds sensitivity to the modelling by removing the inter-individual variation in *Ṁ*O_2peak_. The individual DO_AAS-50_ values can then be pooled to generate mean values for statistical comparison, say across species, at different temperatures for the same species and after acclimation to different hypoxic conditions that might represent future scenarios. Encouragingly, a 21% difference in DO_AAS-50_ was discovered for hypoxia- and normoxia-acclimated sea bass (38 vs. 48 % sat., respectively; *p* = 0.0031; Figure 4a; [51]). Moreover, *Ṁ*O_2peak_ at DO_AAS-50_ was not statistically different from *Ṁ*O_2max_ of sea bass acclimated to and tested at the same 50% DO (*t*-test: *p* = 0.065; Figure 4b; [51]).

In conclusion, we believe individual LOC performance curves hold promise for a broader application in physiology, ecology and evolution. An LOC performance curve can be generated in about three hours, with replication limited only by the number of individual respirometry chambers that are run simultaneously. Moreover, if DO is converted to a PO_2_, the PO_2AAS-50_ can be directly compared with P_crit_ and P_50_. Such comparisons could then provide novel mechanistic insights into hypoxic performance.

## 4. A Benefit of Characterizing Aerobic and Glycolytic Capacities on the Same Individuals

If respiratory phenotyping with maximum aerobic capacity, DO_AAS-50_ and O_2crit_ are performed on the same set of individuals, new analysis is possible beyond simple comparisons. One possibility is a regression analysis to ask if the various indices of aerobic and glycolytic capacities are correlated at the individual level, especially in athletic fish species. This possibility exists because most athletic fish species need a high aerobic capacity (i.e., oxidative phosphorylation) to sustain a high demand in locomotion, while short bouts of peak activity are greatly subsidized by glycolytic capacity (i.e., substrate-level phosphorylation). Some marine fishes even migrate vertically and diurnally between pelagic normoxic zones and coastal O_2_-minimum zones to actively forage in hypoxic habitats [92,93]. Yet, we are not aware of a comparative exploration to understand the correlations of whole-organism aerobic and glycolytic capacities in athletic fish species.

The advantage of an individual-based approach is that it considers the very variation that natural selection acts on. Furthermore, a standardized testing approach used in all the studies can greatly reduce the potential of including measurement discrepancies, which is possible when the broad comparison involves different test conditions. The Integrated Respiratory Assessment Protocol (IRAP), which measures 13 aerobic and anaerobic traits [78], has recently been used broadly to respiratory phenotype three athletic fish species in North America and Europe: rainbow trout in California and British Columbia, Atlantic salmon in Norway and British Columbia, and European sea bass in France.

**Figure 5:**
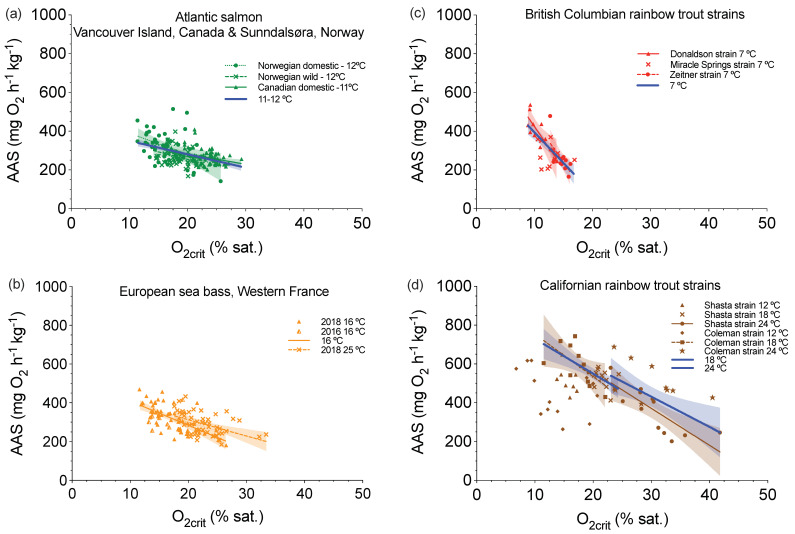
Linear regression (mean ± 95% C.I.) for critical dissolved oxygen (O_2crit_) and absolute aerobic scope (AAS) in three athletic fish species. The two hallmarks of hypoxia performance curve, O_2crit_ and AAS, were measured on the same set of individuals for Atlantic salmon (*Salmo salar*) at Vancouver Island, Canada, and Sunndalsøra, Norway (**a**), European sea bass (*Dicentrarchus labrax*), Western France, (**b**) rainbow trout (*Oncorhynchus mykiss*) strains at British Columbia, Canada (**c**) and California, United States (**d**). Geographic locations are separated by different colours. Testing groups are separated by different symbols within a species. Significant linear regressions are labeled (lines) for Donaldson strain at 7 °C (y = −43.8x + 863.1, *p* = 0.0065, R^2^ = 0.63), Zeitner strain at 7 °C (y = −43.4x+ 889.7, *p* = 0.016, R^2^ = 0.45), Shasta strain at 24 °C (y = −19.0x + 940.7, *p* = 0.0019, R^2^ = 0.64), Coleman strain at 18 °C (y = −21.8x + 971.9, *p* = 0.042, R^2^ = 0.38), European sea bass at 16 °C, pooled 2016 and 2018 (y = −12.5x + 534.4, *p* < 0.0001, R^2^ = 0.38), Norwegian domestic Atlantic salmon at 12 °C (y = −7.8x + 409.8, *p* = 0.013, R^2^ = 0.14), Norwegian wild Atlantic salmon at 12 °C (y = −11.2x + 502.1, *p* = 0.0032, R^2^ = 0.18), and Canadian domestic Atlantic salmon at 11 °C (y = −5.2x + 385.2, *p* = 0.0012, R^2^ = 0.13). The figure legends without a regression line mean no relationship in some testing groups: Miracle Spring strain at 11 °C (y = −6.3x + 358.4, *p* = 0.58, R^2^ = 0.63), Shasta strain at 12 °C (Y = −5.7x + 614.7, *p* = 0.49, R^2^ = 0.062), Shasta strain at 18 °C (y = −14.0x + 826.5, *p* = 0.19, R^2^ = 0.15), Coleman strain at 12 °C (y = −10.1x + 585.7, *p* = 0.18, R^2^ = 0.16), Coleman strain at 24 °C (y = −10.1x + 816.9, *p* = 0.13, R^2^ = 0.30). Furthermore, the species tested under the similar temperature (± 1 °C) are pooled (significant regression lines were denoted) for British Columbia rainbow trout at 7 °C (y = −5.2x + 385.2, *p* = 0.0012, R^2^ = 0.13), Atlantic salmon at 11–12 °C (y = −6.9x + 419.0, *p* < 0.0001, R^2^ = 0.22), European sea bass at 16 °C (y = −13.8x + 561.0, *p* < 0.0001, R^2^ = 0.48) and 25 °C (y = −8.3x + 478.6, *p* = 0.0004, R^2^ = 0.24), and Californian rainbow trout at 18 °C (y = −18.0x + 909.8, *p* = 0.0003, R^2^ = 0.45) and 24 °C (y = −15.6x + 898.4, *p* = 0.0051, R^2^ = 0.34), but not 12 °C (y = −4.9x + 551.4, *p* = 0.37, R^2^ = 0.038).

One relationship that transcended all the species we examined was that O_2crit_ (difference: 1.8–6.1 folds, cv = 17.6–34.5%) and AAS (differences: 2.5–3.6 folds, cv = 20.6–31.1%) were quantitatively related at the individual level (y = −4.3x + 412.2, *p* < 0.0001, R^2^ = 0.04, when all data were pooled). Thus, individuals that can better extract O_2_ in hypoxia (a lower O_2crit_) also tended to have a higher aerobic capacity in normoxia (Figure 5). Although significant correlations (*p* ≤ 0.042, 0.13 ≤ R^2^ ≤ 0.63 for groups of significant relationships) did not necessarily exist for all testing temperature in every population that we considered (e.g., Californian rainbow trout strains tested at 12 °C), the relationships were remarkably consistent when a species was tested at the same temperature (*p* ≤ 0.0051, 0.22 ≤ R^2^ ≤ 0.48). In fact, a warmer acclimation temperature tended to shift the regression lines to the right (Figure 5b,d for sea bass and rainbow trout).

Acclimation to hypoxia will be one of the keys capabilities for fish species to succeed in a more hypoxic future. We know that fish have an incredible hypoxia acclimation capacity. For example, hypoxia acclimation increases *Ṁ*O_2max_ and aerobic scope in rainbow trout and Atlantic cod (*Gadus morhua*) [80,94], by improving O_2_ uptake at the gills, O_2_ delivered to the mitochondria and thereby the capacity to aerobically meet the metabolic needs [94,95]. The increased aerobic capacity in normoxia due to hypoxia acclimation likely attributes to the enhanced O_2_ transport cascade as a beneficial carryover effect. As illustrated by the LOC curve, fish in hypoxia cannot fully sustain an aerobic capacity. Thus, an enhanced O_2_ transport cascade likely contributes to an improved defence for aerobic capacity in moderate hypoxia. In this regard, hypoxia acclimation led to a left-shifted LOC curve and DO_AAS-50_ (Figure 4), showing fish can maintain the same *Ṁ*O_2peak_ to a lower DO, which should also translate to a better O_2_ extraction in hypoxia (i.e., a decline in O_2crit_). Indeed, hypoxia acclimation leads to a decrease of O_2crit_ in epaulette sharks (*Hemiscyllium ocellatum*) [68], sailfin molly (*Poecilia latipinna*) [96], goldfish [97], Atlantic salmon (*Salmo salar*) [98] and snapper (*Pagrus auratus*) [99]. Still, future work is warranted to explore whether the phenotypic plasticity extends beyond the tolerance traits. In this regard, the LOC performance curve will advance a holistic characterization of the individual variations and plasticity for a hypoxia performance curve to quantify the sub-lethal effects. Although mortality events strip out the unfitted individuals, the tipping point that sets off a chain reaction of cascade events leading to mortality is the mechanisms of evolution at work, of which we have very little understanding.

## 5. Conclusions

To date, research on how fishes will live in a more hypoxic future mostly focused on tolerance traits rather than the sublethal effects at the moderate ambient O_2_ levels. The reduced O_2_ availability in the water suppressed the O_2_ need for fish. Such idea is dispersed in the literature as a conceptual framework for a whole-organism hypoxic performance curve [10,11,36], experimental work of LOC performance curve for a fish population [53] and biochemistry concept of scope for survival [55]. By organically integrating these ideas as a tangible individual LOC performance curve, we will better understand the sublethal effects of a continuous shift of meeting the metabolic demands from sustained aerobic capacity to the time-limited anaerobic capacity as water O_2_ content declines. Among these, a mid-point on the LOC performance curve (DO_AAS-50_) provides a comparative indicator for the individual differences in coping with the sublethal effects, where aerobic capacity is clearly suppressed, and substrate-level phosphorylation can be engaged. As the measurements of the hypoxia performance curve are carried out in high throughput aquatic respirometry systems (8, 16 and even 32 fish being run simultaneously), fish biologists are well poised to understand the standing individual variation of the hypoxic performance, one of the key pillars to better bridge physiology and ecology.

## Abbreviations

AAS	absolute aerobic scope
AOD	accumulated O_2_ deficit
CaO_2_	arterial blood O_2_ content
C_crit_	critical O_2_ concentration
C_v_O_2_	venous O_2_ content
DO	dissolved O_2_ level
DO_AAS-50_	the water-dissolved O_2_ that supports 50% of the individual’s AAS
ILOS	incipient lethal O_2_ saturation
LOC	limiting-O_2_ concentration
MMR	maximum metabolic rate
*Ṁ*O_2max_	maximum O_2_ uptake
*Ṁ*O_2_	O_2_ uptake
*Ṁ*O_2peak_	peak O_2_ uptake
O_2_	oxygen
O_2crit_	critical dissolved O_2_
P_50_	a partial O_2_ pressure where hemoglobin is 50% saturated with O_2_
P_crit_	critical O_2_ tension
PO_2_	partial pressure of O_2_, or O_2_ tension
RMR	routine metabolic rate
SMR	standard metabolic rate
*U*_crit_	critical swimming speed
